# Analyzing the key performance indicators of circular supply chains by hybrid fuzzy cognitive mapping and Fuzzy DEMATEL: evidence from healthcare sector

**DOI:** 10.1007/s10668-022-02535-9

**Published:** 2022-07-04

**Authors:** Asana Hosseini Dolatabad, Hannan Amoozad Mahdiraji, Ali Zamani Babgohari, Jose Arturo Garza-Reyes, Ahad Ai

**Affiliations:** 1 Faculty of Management, University of Tehran, Tehran, Iran; 2 School of Business, University of Leicester, Leicester, UK; 3 Centre for Supply Chain Improvement, University of Derby, Derby, UK; 4 College of Engineering, Lawrence Technological University, Michigan, United States

**Keywords:** Healthcare performance measurement, Healthcare key performance indicators, Circular supply chain, Fuzzy cognitive map, Fuzzy DEMATEL

## Abstract

This study presents a multi-layer fuzzy-based decision-making approach to enhance the hospital Circular Supply Chain (CSC) performance by focusing on intensive care units (ICU) via key performance indicators analysis. In this regard, a Systematic Literature Review (SLR) and Institution Fuzzy Delphi (IFD) are employed to extract the relevant and prominent KPIs. After, a hybrid Fuzzy Cognitive Mapping (FCM) and Fuzzy Decision Making Trial and Evaluation Laboratory (FDEMATEL) have been applied to illustrate a conceptual framework for the CSC performance management of the healthcare sector in the emerging economy of Iran. As a result, eight critical indicators emanated from the SLR-IFD approach. Furthermore, sixteen relationships amongst the performance indicators were identified via hybrid FCM-FDEMATEL. Inventory availability, information availability, innovation, and technology were selected as the most influential indicators. Besides, changing the information technology category, including information availability and Innovation and technology, had the most impact on the performance of the entire CSC. This study attempts to evaluate hospitals’ circular supply chain performance, by designing the circular evaluation framework. Hospital managers can use the results of this research to improve their internal circular supply chain performances in the intensive care units by understanding the different scenarios.

## Introduction

During the last decades, scholars have received high attention to improving the healthcare system and addressing related challenges. Fast mutations in medical sciences put health care issues under intense pressure (Lenin, 2014). Hence, various entities in healthcare systems, e.g. hospitals are obliged to pay more attention to the manufacturing and maintenance processes to reduce monetary and non-monetary costs, improve quality of service, safety, optimize service processes, and personnel satisfaction by implementing effective initiatives (Moons et al., 2019a, 2019b). The intensive care unit (ICU) is defined as an important part of the hospital, which provides high-quality care for patients with critical illnesses. The primary purpose of the ICU is to provide a comfortable staying and safe environment for the patients, ICU staff, and visitors (Luongo et al., 2016). As critical patients have been cared for in ICUs, small improvements on a large scale can save more lives and decrease mortality.

Product standardization and Supply Chain Management (SCM) can be considered good targets to improve ICU efficiency (Rothstein & Raval, 2018). SCM can be successful if effective coordination and integration amongst various supply chain components such as suppliers, manufacturers, distributors, and retailers (Lenin, 2014). For this aim, a circular supply chain (CSC) results in zero waste, based on improving the collaboration among stakeholders and integration of surrounding industries and environmental factors (Farooque et al., 2019). Based on the recycling policy, reuse and reduction are vital features of CSC (Khan & Ali, 2022). Therefore, CSCM strategies are appropriate procedures for gaining operational advantages by considering structural adjustments in the companies (Tseng et al., 2021). The global transformation from the linear concept to the circular illustrates great opportunities to decrease waste services and production (Chioatto & Sospiro, 2022). Hospitals are following the procedures for increasing the visibility of CSC processes resulting in lower costs and waste. The most important principle in the hospitals is patient care; nevertheless, CSC processes are vital for providing safety, availability, visibility, and affordability of supplies (Moons et al., 2019a, 2019b).

Gaining CSC advantages is a significant challenge for supply chain managers, as CSC guides them to increase profitability, and efficiency and decrease negative social, environmental, and economic impacts (Farooque et al., 2019). Thus, strategic and operational performance indicators based on the CSC infrastructures are essential for measuring hospital performances (Tseng et al., 2021). Many researchers employed performance indicators to assess hospital performance for improving healthcare management (Christiansen & Vrangbæk, 2018). It is urgent to identify a limited number of key performance indicators (KPIs) (Núñez et al., 2018) as improving all indicators is not possible. KPIs are used to evaluate institutions by setting aims, supporting plans, monitoring outcomes, and reporting hospitals’ achievements and consequences (NAR et al., 2021).

While researchers have paid much attention to healthcare issues, there is little effort to solve problems related to the healthcare circular supply chain (HCSC). Furthermore, in the vast majority of cases, the scholars illustrated healthcare KPIs regardless of the relationships existing among them in the CSC simultaneously. Hence, this study is one of the first efforts to fill this gap by providing a structured framework for analysing the KPIs of ICUs in a circular healthcare supply chain to improve the internal CSC processes. In fact, given the gaps in the literature, the research questions to gain the main research’s purpose can be formulated as (i) what are the most critical healthcare KPIs identified from the literature review? (ii) which KPIs are more important relatively? (iii); what are the casual relationships amongst the selected KPIs?

This study first classifies a set of CSC KPIs based on the frequency of the literature review. In addition, this study tries to develop the pervious used methodologies by considering uncertainty in data collection and interdependence among the CSC indicators, simultaneously. To overcome the large number of KPIs, the Intuitionistic Fuzzy Delphi (IFD) approach was applied by removing the redundant KPIs based on the experts’ vision (Tseng et al., 2020). This method is appropriate, particularly when dealing with the challenge of indicators’ interactions. Furthermore, intuitionistic fuzzy sets are used to address the decision-making processes’ uncertainties and obtain experts’ opinions (Lotfi et al., 2018; Tirkolaee & Aydin, 2022). It should be noted that this method can aggregate the experts’ opinions and use the threshold to decide on the final list of CSC indicators. Hence, this manuscript presents a fuzzy decisions support approach using multi-attribute decision-making (MADM) methods to analyse the healthcare KPIs for CSC improvement and gain sustainable competitive advantage. Moreover, FCM is applied as a useful decision-making tool in the KPIs analysis of healthcare CSC. FCM helps managers to reduce risk management and provide corrective procedures for improving the system performance by considering cognitive mechanisms (Bevilacqua et al., 2018). Also, displaying the causal relationships that may happen in the system and decreasing the dependency on experts’ visions are other features of this method (Bakhtavar & Shirvand, 2019). Furthermore, the Fuzzy DEMATEL method is used to remove redundant interdependence relationships and provides potential interactions and the weights of the KPIs (Tseng et al., 2020). In other words, this study illustrates the interrelationships among KPIs in the CSC and suggests a cause-and-effect framework to enhance managerial insights in the healthcare industry. Strategic organizational managers and professionals will be able to concentrate on certain KPIs to enhance the organization's overall CSC performance. The proposed model demonstrates insights into the possible framework of KPIs in the healthcare industry, particularly in ICUs.

The remaining part of this paper is organized as follows. The literature of related works is reviewed in Sect. 2 by considering the healthcare performance management and applications of MCDM in hospitals. In Sect. 3, the methodology including IFD, FCM, and FDEMATEL is expressed for KPI analysis. The results are summarized and discussed in Sect. 4. In Sect. 5, the conclusion, a summary of the study, discussing the managerial implications of the present work, and outlining future directions are considered.

## Literature review

The circular supply chain is described by integrated circular economics (CE) into SCM (Aiassi et al., 2020). The global issues are moving towards CSCM due to increasing concern about world population and waste (Khan & Ali, 2022; Tirkolaee & Torkayesh, 2022). Nevertheless, there is limited research adopted for studying the internal hospital performance in the CSC. Hospital management, which is a complex system with several organizational units and various processes, is unique and different from other industries. (Bélanger et al., 2018). In this regard, Jain et al. developed a strategic framework for evaluating the CSCM to reduce the cost and ensure a competitive advantage (Jain et al., 2018). Farooque et al. reviewed 261 articles based on the current state of CSCM and defined a definition of CSCM (Farooque et al., 2019). Some scholars applied content analysis methodology or reviewed the CSC literature (Lahane et al., 2020) only. There has been high attention among researchers for performance management in the healthcare sector (Jiang et al., 2020). The increased competitive pressure of COVID-19 has forced hospitals to revise their evaluating management system (Ghadir et al., 2022; Lotfi, Kargar, Gharehbaghi, et al., 2021a). There are several important issues for the hospital circular supply chain such as supply chain inefficiencies, redundant administrative costs, and unsuitable care, waste. (Lotfi et al., 2022). Therefore, an effective CSCM can influence positively hospitals (Toba et al., 2008). Choosing significant performance indicators, are important to have an effective framework with a clear definition of performance evaluation (Carlucci, 2010). Managers are following appropriate procedures to evaluate the processes and identify improvement opportunities (Supeekit et al., 2016). Volland et al. reviewed literature based on materials logistics in a healthcare system specifically, hospitals (Volland et al., 2017). Cinaroglu and Baser pursued the relationship between health outcome indicators and effectiveness in Turkey to improve the quality and understanding of the relationship among key performance measures by using a path analytic model (Cinaroglu & Baser, 2018). Some scholars developed a set of operational healthcare logistics performance indicators by using The Analytical Network Process (ANP) and discussed the interdependencies between operational and national performance (Kritchanchai et al., 2018). Moreover, some researchers used the balanced scorecard for evaluating Efficiency that is feasible and relevant in private hospitals (Behrouzi & Ma’aram, 2019). Others measured KPIs of the internal supply chain in the hospital for improving the logistic activities based on the inventory management and distribution activities(Moons et al., 2019a, 2019b). They mainly focused on relevant indicators regarding improving hospital supply chain processes (Moons et al., 2019a, 2019b). Furthermore, some scholars determined features of performance management systems in the industry 4.0 era and provided non-technological management innovations as a superior factor (Robert et al., 2020). Jiang developed in 2020 the DEMATEL method by using linguistic Z-numbers to identify KPIs based on the cause and effect relationships of performance indicators (Jiang et al., 2020). Recently, some researchers provided a set of KPIs for evaluating the healthcare sector during a pandemic crisis (Burlea-Schiopoiu & Ferhati, 2021). Some KPIs used in previous studies are demonstrated in Table 1.

**Table 1:** Review of KPIs in the healthcare sectorɱɱ

	Iv	IAv	IAC	IC	IU	PS	UD	DA	DC	INAC	INAV	PI	EU	RT	ICR	DF	RP	INCO	ST	PSA	MR	QB	INT	SV	ES	ET	ST	AHS	HR
De Pourcq et al., (2015)		✓		✓		✓	✓										✓	✓		✓					✓			✓	✓
Hoeur and Kritchanchai (2015)	✓	✓	✓	✓	✓		✓		✓	✓	✓	✓	✓	✓				✓	✓									✓	
Carrus et al., (2015)			✓	✓		✓			✓	✓	✓			✓		✓	✓	✓	✓				✓			✓		✓	
Feibert and Jacobsen (2015)		✓					✓				✓		✓	✓			✓		✓				✓		✓				
Fong et al., (2016)		✓		✓	✓	✓							✓						✓										
Supeekit et al., (2016)		✓		✓		✓			✓				✓	✓			✓	✓										✓	
Rahimi et al., (2017)		✓		✓	✓	✓			✓		✓				✓		✓		✓	✓	✓		✓		✓	✓	✓	✓	
Si et al., (2017)	✓	✓		✓	✓	✓			✓	✓	✓						✓	✓		✓	✓				✓	✓		✓	✓
Gu and Itoh (2018)		✓		✓	✓	✓			✓		✓						✓	✓		✓	✓				✓	✓	✓	✓	
Núñez et al., (2018)	✓	✓	✓	✓	✓	✓			✓	✓	✓			✓	✓		✓	✓	✓	✓			✓		✓		✓	✓	✓
El Mokrini et al., (2018)		✓		✓					✓									✓		✓								✓	
Kritchanchai et al., (2018)	✓	✓	✓				✓	✓		✓	✓	✓	✓	✓									✓					✓	
Moons and et al., (2019a, 2019b)	✓	✓	✓	✓	✓			✓	✓		✓				✓		✓		✓										
Hristov and Chirico (2019)		✓		✓	✓					✓	✓	✓		✓				✓	✓	✓			✓		✓	✓			
Behrouzi & Ma’aram (2019)	✓	✓		✓	✓	✓					✓			✓	✓	✓	✓	✓		✓	✓			✓	✓	✓	✓	✓	✓
Amos et al., (2020)				✓		✓	✓		✓					✓	✓		✓	✓	✓	✓			✓		✓	✓	✓		
Jiang et al., (2020)	✓	✓		✓	✓	✓				✓	✓						✓			✓	✓				✓	✓		✓	✓
J. Lai and Yuen (2020)		✓	✓	✓		✓			✓	✓	✓			✓	✓		✓		✓	✓		✓	✓					✓	
Pishnamazzadeh et al., (2020)		✓								✓	✓						✓			✓					✓			✓	
Amos et al., (2021)						✓			✓				✓	✓			✓		✓	✓			✓		✓	✓	✓		
Neri et al., (2021)		✓		✓	✓	✓		✓	✓		✓			✓		✓	✓	✓	✓	✓			✓	✓	✓	✓			
Burlea-Schiopoiu and Ferhati (2021)	✓	✓		✓	✓	✓	✓	✓	✓	✓	✓						✓	✓	✓	✓	✓	✓	✓					✓	✓
J. H. K. Lai et al., (2022)		✓		✓					✓	✓	✓						✓		✓			✓	✓						
**This Study**		✓				✓					✓						✓			✓			✓		✓			✓	

^Inventory Visibility (IV); Inventory Availability (IAV); inventory accuracy (IAC); inventory cost (IC); Inventory usage (IU) Patient safety (delays, errors) (PS); Urgent Delivery (UD); Delivery accuracy (DA); Distribution cost (DC); Information Accuracy (INAC); Information Availability (INAV); Product Identification (PI); ease of use (EU); reliable tracking (RT); inventory critically (ICR); delivery frequency (DF); responsibility (RP); information cost (INCO); Standardization (ST); Patient satisfaction (PSA); Mortality Rate (MR) Quality of the building (QB); Innovation and technology (INT); service variety (SV); Employee satisfaction (ES); employee turnover (ET); Staff training (ST); Average hospital stay (AHS); Hospital readmission rate (HR)^

Some researchers applied the Multiple Criteria Decision Making (MCDM) approaches in their healthcare studies and hospitals (Jiang et al., 2020). MCDM methods have been increasingly employed in the healthcare system to support SCM decisions (Adunlin et al., 2015). Using MCDM is proposed because of using both quantitative and qualitative data, calculates contradictory aims, and makes the decision process more clear, efficient, and logical (Adunlin et al., 2015). In this way, Kritchanchai applied the ANP method to develop the hierarchical evaluation of healthcare logistics performances at the operational level (Kritchanchai et al., 2018). Aung in 2019 applied AHP and ANP methods for measuring medical waste management practices of public hospitals in Myanmar (Aung et al., 2019). In addition, Chen developed the interval-valued Pythagorean fuzzy (IVPF) set theory and the VIKOR method for multiple criteria decision analysis for hospital-based post-acute care (Chen, 2019). Recently, some researchers in 2021 selected a suitable location for a hospital during the pandemic by using an integrated method based on Delphi, Best–Worst Method, and interval type-2 fuzzy TOPSIS Technique (Aydin & Seker, 2021). Others identified and prioritized internal and external health care performance indicators by using the Best–Worst Method (Shojaei et al., 2021). Moreover, some researchers prioritized resilience strategies by using an integrated method of AHP and Fuzzy TOPSIS for healthcare supply chains Rehman (ur Rehman & Ali, 2021). Chandra et al. Introduced a framework that the universal immunization program (UIP) in India improves its vaccine supply chain based on sustainable development goals by measuring KPIs based on a balanced scorecard (Chandra & Kumar, 2021).

These days, FCM is used in many fields including healthcare, business, environment, energy, politics, and social sciences (Wang et al., 2019). FCM can be used to depict the behaviour of a physical system by using nodes and edges (Felix et al., 2019). Moreover, in the context of CSC, DEMATEL, Interpretive Structural Modelling (ISM), and Analytic hierarchy process (AHP) methods are the most used techniques. AHP has been extensively used due to its simplicity, but it is not capable of analysing the complex interdependencies among KPIs. DEMATEL and ISM have been found to have an edge over AHP due to their usefulness in capturing interdependencies. In the ISM method, the KPIs are classified into four possible categories, whereas in the DEMATEL method the intensity of impact is captured. However, the limitation of ISM can be overcome by hybrid DEMATEL-FCM methods which are used in this study (Farooque et al., 2020). The other important reason for choosing the FCM method is following a simple mathematical algorithm to derive the suitable control signals and reach the desired values for the system. The value of each concept is calculated based on the relationship of the concept to the other concepts through the connection weights between and the concept’s value (Behrooz et al., 2018).

The above literature review demonstrates that although many efforts have been spent on healthcare performance measurement and improvement, there is still no agreement on KPIs, particularly based on CSC. Current literature misses methodological approaches to evaluate the CSC performance in ICU. Thus, this research aims to describe a list of performance indicators for measuring internal activities based on the hospitals’ CSC goals. The proposed framework ensures an obvious performance definition and aligns objectives with KPIs providing an integrated vision of the ICU based on CSC. Moreover, by using the combination of FCM and FDEMATEL, healthcare managers can understand the most influential and the most impression indicators based on output and input degrees. In fact, by comprehending the indicators relations, healthcare managers can manage logically the indicators, which are in the hot spots and have more influence on others.

## Methodology

**Fig. 1:**
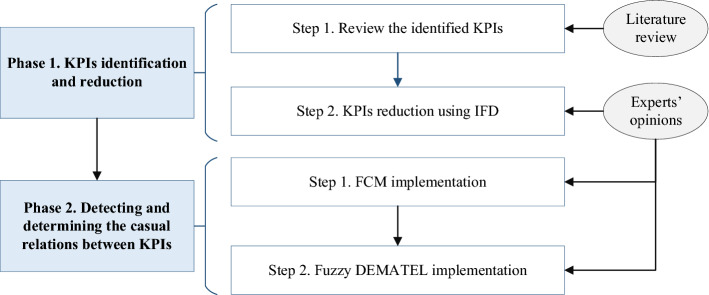
Research framework

The main purpose of this research is to provide an assessment framework based on IFD, FCM, and FDEMATEL to improve hospital processes, particularly in the ICU. The current research focused specifically on overcoming various challenges in implementing hybrid MADM techniques by using appropriately integrated approaches such as IFD, FCM, and FDEMATEL. The numerous KPIs previously reported by scholars have presented a challenge to decision-making processes because the analysis of these KPIs based on committee members’ opinions means that only a limited number of KPIs can be used to ensure sufficient confidence in decisions and their results (Liu et al., 2015). To overcome this challenge, in the first phase, by applying a combination of the literature review and IFD, the identified KPIs were examined and reduced. The IFD method can deal with the fuzziness associated with expert intuition. In various previous studies, triangular, trapezoidal, and Gaussian fuzzy numbers have been used as fuzzy membership functions. In the second phase, the finalized KPIs’ casual relations were determined through two tools, FCM and FDEMATEL to compare the results of both methods to provide a more logical analysis of KPIs. FCM is a modelling approach that follows an approach similar to both human reasoning and human decision-making processes (Nasirzadeh et al., 2020). Furthermore, FDEMATEL helps to uncover the causal interactions among the KPIs based on their cause-and-effect groups (Papageorgiou et al., 2020). (Fig. 1).

The notation list for all methodologies is represented in Table 2.

**Table 2:** Notation list for IFD, FCM, and FDEMATEL

Index	Description	Array or Value
*Part a. IFD notation list*		
A	IFS in a finite set X	−
$\mu (x)$	Membership function	[0,1]
$\vartheta (x)$	Non-Membership function	[0,1]
$\pi (x)$	Hesitation degree	–
L	Number of DMs	16
K	DM kth	$k = \left\{ {1,2,...,L} \right\}$
${D}_{k}$	The intuitionistic fuzzy number for the ranking of $kth$ DM	$\left[ {\mu_{k} ,\vartheta_{k} ,\pi_{k} } \right]$
${\lambda }_{k}$	Weight of kth DM	[0,1]
*Part b. FDEMATEL notation list*		
N	Number of criteria	$n = \left\{ {1,2, \ldots } \right\}$
p	Number of surveyed experts	$p = \left\{ {1,2, \ldots } \right\}$
${\tilde{x }}_{s}$	Fuzzy number (low, med, up)	$\tilde{x}_{s} = \left( {l_{ij} ,m_{ij} ,u_{ij} } \right)$
${\tilde{R }}^{\left(k\right)}$	Normalized Fuzzy matrix $\left( {r_{\ell }^{\left( k \right)} ,r_{m}^{\left( k \right)} ,r_{u}^{\left( k \right)} } \right)$	$k = 1, \ldots ,p$
${\tilde{D }}_{i}$	Impact of criteria	$\left( {i = 1,2, \ldots ,n} \right)$
${\tilde{R }}_{i}$	Degree of influence of criteria	$\left( {j = 1,2, \ldots ,n} \right)$
*Part c. FCM notation list*		
${W}_{ij}$	Weight of criteria	–
f	Threshold function	[0,1]
$i$	Number of criteria	$i = \left\{ {1,2,...,N} \right\}$

### Intuitionistic Fuzzy Delphi (IFD)

The fuzzy Delphi method, which integrates the IFD method and fuzzy theory, was first introduced by (Ouyang & Guo, 2018). In the present research, the data were collected concerning a 5-point Likert scale. The selected method was implemented as suggested by (Hsu et al., 2010). Intuitionistic fuzzy sets (IFSs) have extended to many multi-criteria decision-making methods. IFS was initially introduced by (Atanassov, 2016) as an extension of the conventional FST, which is a proper approach to coping with vagueness (Tirkolaee & Torkayesh, 2022). To define an IFS, let us assume A is an IFS in a finite set X. Then IFS A can be defined as follows.

1 $$ A = \left\{\langle {x,\mu_{A} \left( x \right),v_{A} \left( x \right)\rangle\left| {x \in X} \right.} \right\} $$

where $\mu_{A} \left( x \right):X \to \left[ {0,1} \right]$ is a membership function and $v_{A} \left( x \right):X \to \left[ {0,1} \right]$ is non-membership function, such that $0 \le \mu_{A} \left( x \right) + v_{A} \left( x \right) \le 1$. Compared with classical fuzzy set theory; IFS has a third parameter which is known as hesitation degree or intuitionistic fuzzy index. Suppose that $\pi_{A} \left( x \right)$ is the hesitation degree of whether x belongs to A or not, then $\pi_{A} \left( x \right)$ can be written as ${\pi }_{A}(x)=1-{\mu }_{A}(x)-{v}_{A}(x)$ for every $x\in X$ where $0\le {\pi }_{A}(x)\le 1$. When the value $\pi_{A} \left( x \right)$ is small, information concerning x is more confident. When the value ${\pi }_{A}(x)$ is great, but information regarding x is much more uncertain. The multiplication operator for IFS is given in Eq. 2. Note that A and B are IFSs of set X.

2 $$ A \otimes B = \left\{ {\mu_{A} \left( x \right).\mu_{B} \left( x \right),v_{A} \left( x \right) + v_{B} \left( x \right) - v_{A} \left( x \right).v_{B} \left( x \right)\left| {x \in X} \right.} \right\} $$

Component-wise matrix multiplication is calculated as follows (Atanassov, 2016).

3 $$A^\circ B = \left[ { \langle\min \left\{ {\mu _{A} \left( x \right),\mu _{B} \left( x \right)} \right\},\max \left\{ {v_{A} \left( x \right),v_{B} } \right\}}\rangle \right] $$

The steps of the proposed IFD are presented as follows.

**Step 1**. Define the decision-making problem in detail and circulate the Likert-scale linguistic questionnaire amongst the decision-makers (DMs). Then transfer each expert opinion to IFNs according to Table 3.

**Table 3:** Linguistic terms and membership/non-membership degrees

Linguistic terms	IFNs
Very Important (VI)	(0.90, 0.10)
Important (I)	(0.75, 0.20)
Medium (M)	(0.50, 0.45)
Unimportant (UI)	(0.35, 0.60)
very unimportant (VUI)	(0.10, 0.90)

**Step 2**. Calculate the weights of DMs. Assume that the decision group contains L DMs. The significance of the DMs is considered as linguistic terms indicated in intuitionistic fuzzy numbers. Let ${D}_{k}=\left[{\mu }_{k},{\vartheta }_{k},{\pi }_{k}\right]$ as the intuitionistic fuzzy number for the ranking of $kth$ DM. Then the weight of kth DM can be calculated as follows.

4 $$ \lambda_{k} = \frac{{\left( {\mu_{k} + \pi_{k} \left( {\frac{{\mu_{k} }}{{\mu_{k} + \vartheta_{k} }}} \right)} \right)}}{{\mathop \sum \nolimits_{k = 1}^{l} \left( {\mu_{k} + \pi_{k} \left( {\frac{{\mu_{k} }}{{\mu_{k} + \vartheta_{k} }}} \right)} \right)}}\,{\text{and}}\;\mathop \sum \limits_{k = 1}^{l} \lambda_{k} = 1 $$

**Step 3**. Create the aggregated intuitionistic fuzzy decision matrix according to the DMs opinions. Suppose ${R}^{\left(k\right)}={\left({r}_{ij}^{\left(k\right)}\right)}_{m\times n}$ is an intuitionistic fuzzy decision matrix for each DM. $\lambda =\left\{{\lambda }_{1},{\lambda }_{2},...,{\lambda }_{l}\right\}$ is the weight of each DM and ${\sum }_{k=1}^{l}{\lambda }_{k}=1,{\lambda }_{k}\in \left[\mathrm{0,1}\right]$. In the group decision-making procedure, all the members' opinions are required to be merged into a group decision opinion. To do so, an aggregated intuitionistic fuzzy decision matrix is needed. For this purpose, (Rajaprakash et al., 2022) proposed an Intuitionistic Fuzzy Weighted Averaging (IFWA) operator to aggregate the group decisions as follows.

5 $$ \begin{aligned} R = & (r_{ij} )_{m \times n} \\ r_{ij} = & IFWA_{\lambda } \left( {r_{ij}^{\left( 1 \right)} ,r_{ij}^{\left( 2 \right)} ,...,r_{ij}^{\left( l \right)} } \right) = \lambda_{1} r_{ij}^{\left( 1 \right)} \oplus \lambda_{2} r_{ij}^{\left( 2 \right)} \oplus ... \oplus \lambda_{l} r_{ij}^{\left( l \right)} \\ = & \left[ {1 - \mathop \prod \limits_{k = 1}^{l} \left( {1 - \mu_{ij}^{\left( k \right)} } \right)^{\lambda k} ,\mathop \prod \limits_{k = 1}^{l} \left( {\vartheta_{ij}^{\left( k \right)} } \right)^{\lambda k} ,\mathop \prod \limits_{k = 1}^{l} \left( {1 - \mu_{ij}^{\left( k \right)} } \right)^{\lambda k} - \mathop \prod \limits_{k = 1}^{l} \left( {\vartheta_{ij}^{\left( k \right)} } \right)^{\lambda k} } \right] \\ \end{aligned} $$

**Step 4**. Determine the weights of KPIs. $W$ is a set of significant grades. To calculate W, every DM opinion regarding the significance of every KPI is required to be merged. Let us assume ${w}_{ij}=\left[{\mu }_{j}^{(k)},{\vartheta }_{j}^{(k)},{\pi }_{j}^{(k)}\right]$ as an IFN given to KPI X_j_ by the kth DM. The weights of the KPIs in the CSC are calculated by using the IFWA following operator.

6 $$ \begin{aligned} w_{ij} = & IFWA_{\lambda } \left( {w_{j}^{\left( 1 \right)} ,w_{j}^{\left( 2 \right)} , \ldots ,w_{j}^{\left( l \right)} } \right) \\ = & \lambda_{1} w_{j}^{\left( 1 \right)} \oplus \lambda_{2} w_{j}^{\left( 2 \right)} \oplus ... \oplus \lambda_{l} w_{j}^{\left( l \right)} \\ = & \left[ {1 - \mathop \prod \limits_{k = 1}^{l} \left( {1 - \mu_{j}^{\left( k \right)} } \right)^{\lambda k} ,\mathop \prod \limits_{k = 1}^{l} \left( {\vartheta_{j}^{\left( k \right)} } \right)^{\lambda k} ,\mathop \prod \limits_{k = 1}^{l} \left( {1 - \mu_{j}^{\left( k \right)} } \right)^{\lambda k} - \mathop \prod \limits_{k = 1}^{l} \left( {\vartheta_{j}^{\left( k \right)} } \right)^{\lambda k} } \right] \\ W = & \left[ {w_{1} ,w_{2} ,...,w_{j} } \right] \\ \end{aligned} $$

### Fuzzy DEMATEL

FDEMATEL procedure for investigating interrelationships between CSC KPIs is described as follows (Gölcük & Baykasoʇlu, 2016; Heidary Dahooie et al., 2021). In the first step called the data gathering, using a DEMATEL questionnaire the data required were collected through the contributions of experts and professionals of the healthcare system. Then, the DMs opinion is transferred to triangular fuzzy values according to Table 4. The experts are asked to determine the impact of each indicator on the others based on the pairwise comparison.

**Table 4:** The triangular Fuzzy values used for linguistic terms (Liou et al., 2008)

Linguistic terms	Fuzzy Number (l,m,u)
Very high influence	(0.75, 1.00, 1.00)
High influence	(0.50, 0.75, 1.00)
Low influence	(0.25, 0.50, 0.75)
Very low influence	(0.00, 0.25, 0.50)
No influence	(0.00, 0.00, 0.25)

**Step 2**. Two direct relation fuzzy matrixes ${x}_{ij} \forall k=1,\dots ,p$ are initially considered, in which ${X}_{ij}$ is a $(n\times n)$ matrix, *n* represents the number of criteria (i.e. KPIs) and *p* is the number of surveyed experts. The committee is asked to make sets of pairwise comparisons in terms of the inter influence of KPIs through consensus. The matrix ${\tilde{X }}_{s}$, which ${\tilde{x }}_{s}=\left({l}_{ij},{m}_{ij},{u}_{ij}\right)$, is denoted as the degree to which they believe KPI $i$ affects KPI $j$. The linguistic terms used to generate the ${\tilde{X }}_{s}$ matrix are shown in Table 4.

**Step 3**. Calculate the average direct relation matrix based on Eq. 7.

7 $$ \tilde{x}_{ij} = \frac{{\mathop \sum \nolimits_{k = 1}^{p} \tilde{x}_{ij}^{\left( k \right)} }}{p} $$

**Step 4**. Normalize the average direct relation matrixes based on Eqs. 8–10.

8 $$ \tilde{x}_{ij}^{\left( k \right)} = \frac{{\tilde{z}_{ij}^{\left( k \right)} }}{{\tilde{R}^{\left( k \right)} }} = \left( {\frac{{\tilde{z}_{ij}^{\left( k \right)} ,\ell }}{{r_{\ell }^{\left( k \right)} }},\frac{{\tilde{z}_{ij}^{\left( k \right)} ,m}}{{r_{m}^{\left( k \right)} }},\frac{{\tilde{z}_{ij}^{\left( k \right)} ,u}}{{r_{u}^{\left( k \right)} }}} \right) $$

9 $$ \tilde{R}^{\left( k \right)} = \left( {r_{\ell }^{\left( k \right)} ,r_{m}^{\left( k \right)} ,r_{u}^{\left( k \right)} } \right),k = 1, \ldots ,p $$

10 $$ r_{s}^{\left( k \right)} = max\left[ {\mathop \sum \limits_{j = 1}^{n} Z_{ij,s}^{\left( k \right)} } \right]\forall s = 1,m,n $$

**Step 5**. Calculate the total-relationship fuzzy matrix $\tilde{T }$ using Eq. 11.

11 $$ \tilde{T} = \mathop {lim}\limits_{w \to \infty } \left( {\tilde{X} + \tilde{X}^{2} + ... + \tilde{X}^{w} } \right) = \tilde{X}\left( {I - \tilde{X}} \right)^{ - 1} $$

**Step 6**. Obtain the sum of rows and columns of the sub-matrixes ${T}_{\mathcal{l}}$, ${T}_{m}$, and ${T}_{u}$ denoted by the fuzzy numbers ${\tilde{D }}_{i}$ and ${\tilde{R }}_{i}$, respectively, with Eqs. 12 and 13.

12 $$ \tilde{D}_{i} = \mathop \sum \limits_{j = 1}^{n} \tilde{t}_{ij} \left( {i = 1,2, \ldots ,n} \right) $$

13 $$ \tilde{R}_{i} = \mathop \sum \limits_{i = 1}^{n} \tilde{t}_{ij} \left( {j = 1,2, \ldots ,n} \right) $$

**Step 7**. Defuzzify ${\tilde{D }}_{i}$ and ${\tilde{R }}_{i}$ using Eq. 14 and measure the cause-effect (E_j_ = R_j _− D_j_) and the impact of each indicator (P_j_ = D_j_ + R_j_). Positive values of E demonstrate the cause and negative values the effects.

14 $$ Deffuzification point = \left\{ {\begin{array}{*{20}c} {u - \sqrt {\left( {u - \ell } \right)\left( {u - m/2} \right)} } & {u - m > m - \ell } \\ {\sqrt {\left( {u - \ell } \right)\left( {u - m} \right)/2} - \ell } & {u - m < m - \ell } \\ m & {{\text{otherwise}}} \\ \end{array} } \right. $$

### Fuzzy Cognitive Map (FCM)

FCM is usually described as an “oriented graph, which displays the degree of a causal relationship between various factors, where knowledge expressions, in the causal relationship, are expressed by either positive or negative signs and different weights” (Felix et al., 2019). In other words, FCM can be demonstrated as a set of objects and arrows between them that illustrate interrelations. There are negative and positive influences from object A to object B, which negative influence means if the value of object A increases, the value of object B will decrease ($${W}_{ij}<0)$$, also, the positive influence means if the value of object A increase, the value of object B will increase ($${W}_{ij}>0)$$. Interrelation is distinguished based on value (weight) that provides the strength of influence. In addition, $${W}_{ij}=0$$ displays no relation between $${C}_{i}$$ and $${C}_{j}$$ (Kiraz et al., 2020). The concepts $${C}_{1}$$, $${C}_{2}$$, $${C}_{3}$$, …, $${C}_{n}$$ represent A, which is the state vector of the system. When forming the state vector A, it is important to define the period, the A state vector shows the current position in the time given (Papageorgiou et al., 2011). The main components of the cognitive maps include the nodes the arc between and the mark on these arcs. Nodes illustrate concepts that explain the system, arcs display cause-and-effect relationships between concepts that weigh [− 1, + 1] and the sign on the arcs indicates the type of causality between the concepts. For the keeping method, the initial vector and weight matrix should be defined. After creating the system structure, the inference algorithm of FCMs is applied as follows.

**Step 1**. Determining ${A}^{k}$ vector showing the existing system state.

**Step 2**. Applying Eqs. 15 and 16 to obtain ${A}^{(k+1)}$ after a defined time.

15 $$ A_{i}^{{\left( {k + 1} \right)}} = f\left( {\left( {A_{i}^{\left( k \right)} } \right) + \mathop \sum \limits_{j = 1, j \ne 1}^{N} W_{ij} \times (A_{i}^{\left( k \right)} )} \right) $$

where ${A}_{i}^{(k+1)}$ is the value of the concept ${C}_{i}$ at step k + 1, ${A}_{j}^{\left(k\right)}$ is the value of the concept ${C}_{j}$ at step k, W is the interaction matrix f, which is the threshold function, that provides transformation within [0, 1]. Various functions are implemented for transformation. In this paper, the sigmoid function, which ensures that the value of each concept, will pertain to the [0,1] is applied as follows.

16 $$ f\left( x \right) = \frac{1}{{1 + e^{ - \lambda x} }} $$

$\lambda$ defines the slope of the sigmoid function. Value can be changed based on the DMs opinion. In this study, the value is taken as (1) to be closer to linearity. The sigmoid function is more advantageous than other activation functions in reaching extreme values such as 0 or 1 (Morone et al., 2019).

**Step 3**. Obtaining ${A}^{(k+1)}$ as the new vector in the next iteration.

**Step 4**. Repeating steps 2 and 3 until ${A}^{(k+1)}-{A}^{\left(k\right)}<0.001$. The value for ${A}^{(k+1)}$ found in each iteration displays the system state in respect of the values predetermined by the experts (Papageorgiou et al., 2020).

## Findings and results

Based on the literature review and the problem statement, there is little effort to solve problems in the HCSC. The current research focused specifically on overcoming various challenges in implementing hybrid MADM techniques by using approaches such as IFD, FCM and FDEMATEL in an integrated fashion. The numerous KPIs previously reported by scholars have presented a challenge to decision-making processes. To overcome this challenge, by applying a combination of the literature review and IFD, the identified KPIs were examined and reduced. Furthermore, previous studies illustrated healthcare KPIs without focusing on the causal relationships amongst them in the CSC. Hence, this study is one of the first efforts to fill this gap by providing a structuralized framework for analysing the KPIs of ICUs in the circular healthcare supply chain to improve the internal CSC processes. Accordingly, the finalized KPIs’ causal relations were determined through two tools, (i) FCM and (ii) FDEMATEL to compare the results to provide a more logical analysis for KPIs. First, the KPIs identified from LR (29 KPIs) were analysed and screened. Selected KPIs (14 out of 29) were addressed in at least 50% of studied articles and are shown in Table 5 (highlighted KPIs in grey).

**Table 5:** Initial analysis of KPIs

KPI	Frequency	KPI	Frequency
Inventory visibility	38	Delivery frequency	12
Inventory availability	87	Response time	78
Inventory accuracy	26	Information cost	50
Inventory cost	83	Standardization	61
Inventory usage (IU)	52	Patient satisfaction	65
Patient safety (delays, errors)	65	Mortality rate	26
Urgent delivery	17	Quality of the building	13
Delivery accuracy	17	Innovation and improvement	52
Distribution cost	70	Service variety	8
Information accuracy	47	Employee satisfaction	57
Inform Action availability	74	Employee turnover	44
Product identification	13	Staff training	26
Ease of use	26	Average hospital stay	65
Accurate and reliable tracking	52	Hospital readmission rate	25
Inventory critically	31		

Then, the IFD method was used to select the critical KPIs in the healthcare CSC. The data collection process included sixteen online meetings in four different groups of two private hospitals, one public hospital, and one department of industrial engineering based on the healthcare sector in the emerging economy of Iran. All experts had high levels of education and worked in the healthcare and ICU sector for at least four years and were reliable to answer the questionnaires. The first interview for each group was about explaining a general comprehension of performance measurement in the circular supply chain, which took approximately one hour. For this aim, the concepts of CSC in the healthcare sector, key performance indicators, their relationships, and the basics of the surveys were discussed. Finally, the four online meetings, after one week, were conducted to gather the experts’ opinions to define the KPIs and the interrelationships among them. The whole data gathering process took over one month. Eight interviews were held for finalizing the KPIs based on IFD by 14 questions according to the Likert scale, and eight online meetings were held for FCM and FDEMATEL based on the pairwise comparison questionnaire of selected KPIs from IFD. Overall, three questionnaires were provided for IFD, FDEMATEL, and FCM. The expert/DMs profile is presented in Table 6.

**Table 6:** Experts/DMs profile

Gender (M/F)	Job role	Working Experience (years)
M	Hospital Manager	6
M	ICU Doctor	10
F	ICU Doctor	4
F	ICU Nurse	4
F	Hospital Manager	7
M	ICU Doctor	9
F	ICU Doctor	6
M	ICU Nurse	5
M	Hospital Manager	4
M	ICU Doctor	8
F	ICU Doctor	11
F	ICU Nurse	8
M	Professor	15
M	Professor	13
F	Associate Professor	15
M	Associate Professor	14

Then, based on the IFD method (Sect. 3.1), after determining the weight of the experts based on Eq. 4, the value of the IFD Number (IFDN) for each KPI was calculated. In this method, the approval or rejection threshold for indexes is considered to be 4. Table 7 illustrates eight CSC KPIs that were confirmed using the IFD approach.

**Table 7:** Selected KPIs from the IFD approach

KPI	IFDN	Decision	Code	Definition
Inventory availability	4.808	✓	C1	Accessible services and products (Kumar & Rahman, 2014)
Inventory cost	3.549			
Inventory usage (IU)	3.042			
Patient safety	6.557	✓	C2	Provide trusty services and keep patients from errors, infections, and delays (Núñez et al., 2018)
Distribution cost	2.546			
Information availability	5.193	✓	C3	The capacity of information technology to demonstrate accurate data in the whole supply chain (Kritchanchai et al., 2018)
Accurate and reliable tracking	3.485			
Response time	6.369	✓	C4	Deliver the on-time services and products (Amos et al., 2021)
Information cost	3.27			
Standardization	3.55			
Patient satisfaction	6.206	✓	C5	Patients’ expectations of all services, products, transportation, and inventory (Gu & Itoh, 2018)
Innovation and improvement	6.206	✓	C6	Supply chain buildings, facilities, services, and products (Kritchanchai et al., 2018)
Employee satisfaction	4.239	✓	C7	Satisfaction of staff (Gu & Itoh, 2018)
Average hospital stay	5.565	✓	C8	The average time which a patient stays in the hospital (Núñez et al., 2018)

For FCM, the Mental modeller software was used to demonstrate the cause-effect diagram based on the average of experts’ opinions, and then their insights were normalized and organized in the matrix based on the weights of the connections between nodes. The initial matrix of experts’ opinions is presented in Table 8.

**Table 8:** Interactive matrix for FCM

Criteria	C_1_	C_2_	C_3_	C_4_	C_5_	C_6_	C_7_	C_8_
C_1_	0	0.5	0	0.9	0.8	0	0.55	0.65
C_2_	0	0	0	0	1	0	0	0.5
C_3_	0	0.5	0	0.8	0	0.5	0.65	0
C_4_	0	0.9	0	0	1	0	0	0
C_5_	0	0	0	0	0	0	0	0
C_6_	0.65	0.8	0.7	0.85	0	0	0	0
C_7_	0	0.65	0	0.65	0.55	0	0	0
C_8_	0	0.55	0	0	0.8	0	0	0

**Fig. 2:**
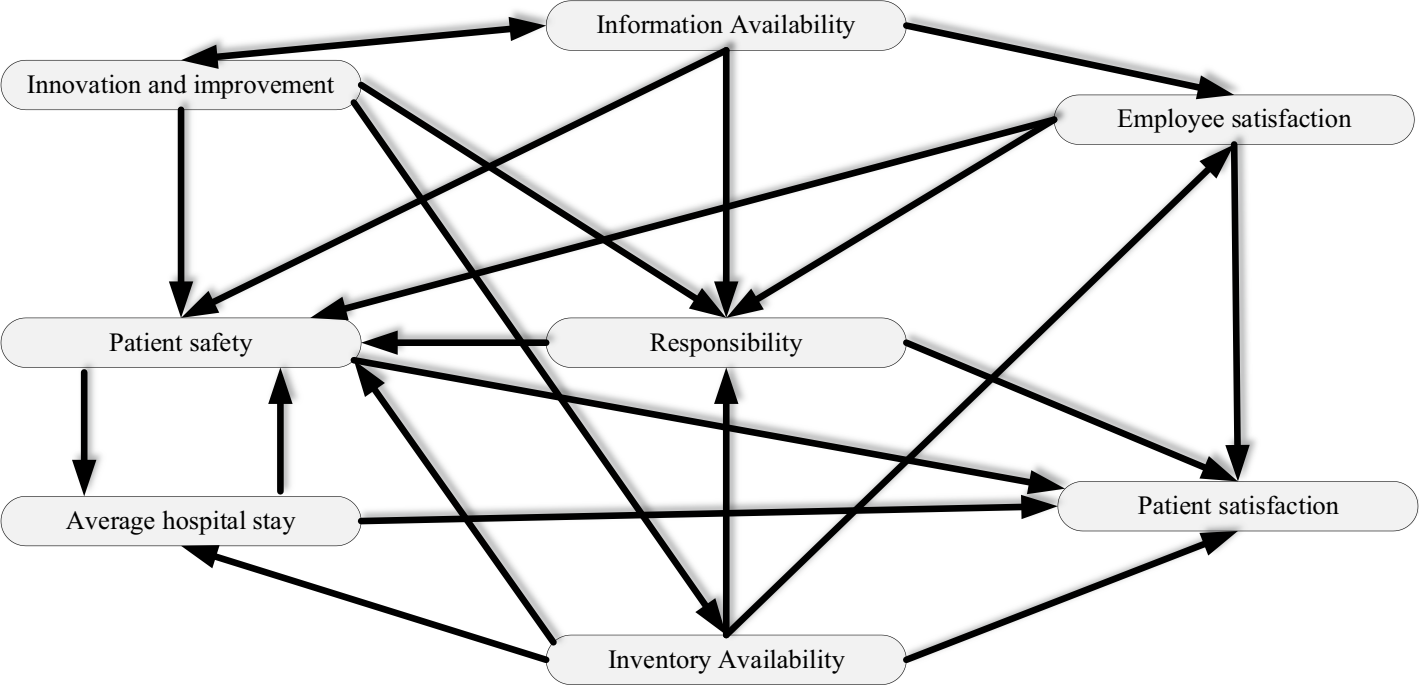
Cause-effect diagram of KPIs by FCM

The result of implementing FCM separately is displayed in Fig. 2, which has eight total components and twenty-two connections. The average connection per component is 2.75.

**Fig. 3:**
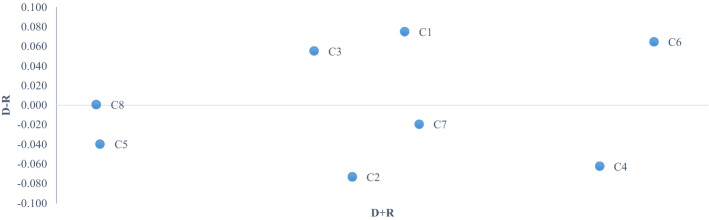
Causal Diagram of the Main KPIs

The FDEMATEL method was applied as suggested by (Dalalah et al., 2011) to compare its results to FCM. FDEMATEL is a structural model used to analyze the causal relationship between complex indicators (KPIs) in various applications, for instance, healthcare management based on CSC (Mavi & Standing, 2018). FDEMATEL models a CSC structural diagram of the system according to the connections amongst the criteria (Hosseini et al., 2021). FDEMATEL is implemented to define the interdependence of the eight KPIs of the healthcare CSC. According to Sect. 3.2, the results of implementing this method are presented in Table 9 and the cause-and-effect values of KPIs are depicted in Fig. 3.

**Table 9:** Total relations matrix

	C_1_	C_2_	C_3_	C_4_	C_5_	C_6_	C_7_	C_8_	$\left({D}_{i}-{R}_{i}\right)$	$\left({D}_{i}+{R}_{i}\right)$
C_1_	0.000	0.011	0.003	0.031	0.015	0.008	0.024	0.027	0.075	0.165
C_2_	0.002	0.000	0.002	0.003	0.009	0.007	0.007	0.006	− 0.073	0.153
C_3_	0.003	0.011	0.000	0.031	0.003	0.023	0.023	0.003	0.055	0.145
C_4_	0.003	0.030	0.003	0.000	0.016	0.008	0.008	0.003	− 0.062	0.207
C_5_	0.002	0.003	0.002	0.003	0.000	0.007	0.007	0.002	− 0.040	0.099
C_6_	0.027	0.028	0.027	0.035	0.004	0.000	0.009	0.003	0.065	0.218
C_7_	0.003	0.019	0.003	0.027	0.005	0.008	0.000	0.003	− 0.020	0.168
C_8_	0.003	0.009	0.003	0.003	0.015	0.007	0.008	0.000	0.000	0.099

**Fig. 4:**
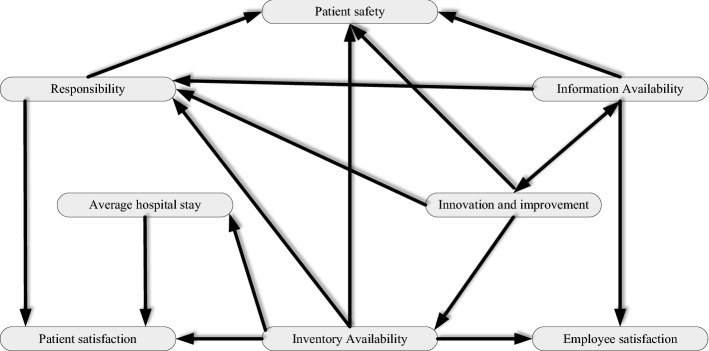
Final model from FCM-FDEMATEL

The total relations matrix is defuzzified and the threshold values are set to 0.0098 (the average value of the total relationship matrix). Therefore, the impact relationship CSC map of FCM was updated according to the FDEMATEL results and shown in Fig. 4.

There are indegree, outdegree, and centrality for each node. Indegree shows the aggregate weights, which have influenced the nodes. Besides, the outdegree displays the summation value which the node affects the other nodes. Eventually, the summation of input and output degrees demonstrates the centrality of each node. The weights of CSC KPIs acquired by the FCM method are presented in the last column by linear normalizing of centrality. Furthermore, Each KPI can be one of the three types of components, driver, receiver, and Ordinary in the healthcare CSC. An ordinary node has both input degree and output degree, a driver has just output degree, and a receiver component deals with just input degree. Based on Fig. 4, there are sixteen connections in the whole system. The average connection per component is 2. Table 10 demonstrates the input, output, and centrality degrees of KPIs according to FCM-FDEMATEL.

**Table 10:** Input, output, and centrality degrees of KPIs

KPI	Indegree	Outdegree	Centrality	Weight
Inventory availability	0.65	3.4	4.05	0.190
Patient safety	2.7	0	2.7	0.127
Information availability	0.7	2.45	3.15	0.148
responsibility	2.55	1	3.55	0.167
Patient satisfaction	1.70	0	1.70	0.080
Innovation and technology	0.5	3	3.5	0.164
Employee satisfaction	1.2	0	1.20	0.056
Average hospital stay	0.65	0.8	1.45	0.068

According to Table 10, there are five ordinary, three-receiver, and no deliver components in the CSC system. Inventory availability (C_1_), information availability (C_3_), responsibility (C_4_), innovation and technology (C_6_), and average hospital stay (C_8_) are ordinary indicators. Patient safety (C_2_), patient satisfaction (C_5_), and employee satisfaction (C_7_) are receiver indicators. Patient safety (C_2_) has the most indegree and inventory availability (C_1_) has the most outdegree among other KPIs. In addition, innovation and improvement (C_6_) have the lowest indegree, and patient satisfaction (C_5_), patient safety (C_2_) and employee satisfaction (C_7_) have the lowest out-degree. Besides, inventory availability (C_1_), responsibility (C_4_) and Innovation and technology (C_6_) have the highest importance based on their centrality, respectively.

## Discussion

As hospital managers are following some evaluating structures, this study is one of the first efforts to provide a structured framework for analysing the KPIs of ICU in the circular healthcare supply chain. Few studies paid attention to evaluating the healthcare circular supply chain. In the traditional supply chain, the focus is on quality, cost, and output; however, the CSC is evaluated by both tangible and intangible indicators (Genovese et al., 2017). These intangible indicators are hidden connections that link seemingly independent components of the supply chain and make them interconnected (Jain et al., 2018). In designing the circular supply chain, traceability, transparency, and trust are demonstrated as the critical features (Centobelli et al., 2021). By considering the FCM-FDEMATEL framework, all eight KPIs were supporting this fact. In the healthcare sector traceability is a vital factor that provides product and service tracking (Sodhi & Tang, 2018), and it can help to improve inventory availability (C_1_), and patient safety (C_2_) based on innovation and improvement (C_6_) and information availability (C_3_). In addition, inventory availability (C_1_) and employee satisfaction (C_7_) were related to transparency, which can increase by raising information availability (C_3_) and understanding the average hospital stay (C_8_). Transparency is defined as easy access to information (Bai & Sarkis, 2020). Furthermore, trust including expectation, reasonable acting, and expected behaviour is a significant feature helping to increase patient satisfaction (C_5_) and patient safety (C_2_) by increasing responsibility (C_4_).

The Sustainable Development Goals (SDGs) are considered the main goal of the international authorities to handle the detrimental effects associated with misleading the environmental, economic, and social policies (Guerra et al., 2021). Therefore, this study illustrated KPIs and their relationships by considering some of the SDGs. For instance, (G3) Good Health and Well-being aim to decrease the number of mortality and achieve a better quality of healthcare services (Çağlar & Gürler, 2021), which was supported by patient safety (C_2_) and average hospital stay (C_8_). Moreover, (G9) industry, innovation, and infrastructure are related to sustainable industrialization, advancement of technological capabilities, and easy access to information (Çağlar & Gürler, 2021). Hence, information availability (C_3_) and innovation and improvement (C_6_) were covered in goal nine. Also, (G12&G17) “Responsible Consumption and Production” and “Partnerships to achieve the goal”, respectively, are relevant to the CSC by generating zero waste and strengthening global cooperation (Tseng et al., 2021). Furthermore, to gain features of the CSC including sustainability and transparency, using new technologies and innovations in the industry 4.0 era is required (Ding, 2018; Torkayesh et al., 2021). For this aim, the innovation and technology (C_6_) indicator supported industry 4.0 technologies. In addition, Healthcare 4.0 allows patients to gain easy access to their information (C_3_) in a responsible manner (C_4_) (Sharma et al., 2019). Therefore, some features of industry 4.0 are used in the HCSC.

**Fig. 5:**
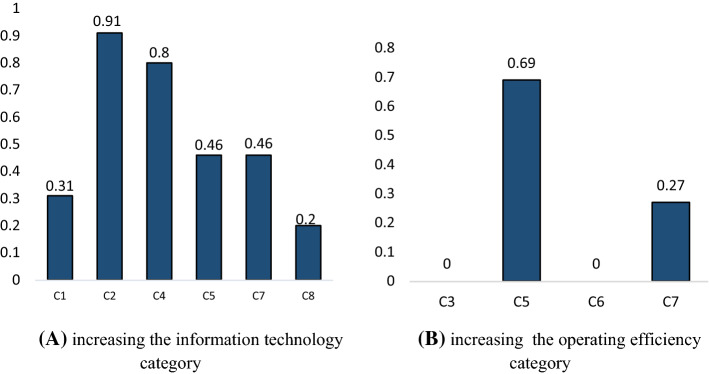
Different Scenarios based on KPIs changes, A) increasing the information technology category, B) increasing the operating efficiency category

This paper attempted to provide some effective subjects for hospital managers to improve their healthcare systems by showing some different scenarios based on analysing the relationships among KPIs. Comparing the artificial scenarios can be helpful for hospital managers to decide what scenarios run better and be more effective in the system (Gray et al., 2013). For this aim, the KPIs were categorized based on three groups, namely: operating efficiency, information technology, and social benefit (Jiang et al., 2020; Kritchanchai et al., 2018). Inventory availability (C_1_), patient safety (C_2_), responsibility (C_4_), and Average hospital stay (C_8_) were considered in the Operating efficiency category. The second group included information availability (C_3_) and Innovation and technology (C_6_). Finally, Patient satisfaction (C_2_) and Employee Satisfaction (C_7_) were categorized into the social benefit group. As hospital managers prefer the simple framework, which is better for understanding, the changes in each category were obtained based on the DEMATEL method, which has lower relationships in its system. Each category implemented a 50% increase on their KPIs, separately. The changes in KPIs based on changing each category are demonstrated in Fig. 5.

Figure 5A) demonstrates that by increasing the information technology indicators, five indicators will change. In fact, by 50% change in the information availability (C_3_) and innovation and technology (C_6_), other KPIs including patient safety (C_2_), responsibility (C_4_), patient satisfaction (C_5_), employee satisfaction (C_7_) and average hospital stay (C_8_) will change more than 20%. Besides, increasing this group of KPIs has a significant impact of more than 80% on patient safety (C_2_), which is the main aim of ICU (Bagshaw et al., 2017) and responsibility (C_4_). On the other hand, the operating efficiency indicators have a significant impact on patient satisfaction (C_5_) by a 69% increase which is shown in Fig. 5B. However, based on further investigations**,** the social benefit KPIs have no impact on other indicators since both patient satisfaction (C_5_) and employee satisfaction (C_7_) were receiver indicators. The results for increasing each group were the same as decreasing them, although in the opposite direction. As a result, changes in the information technology category have more influence on the entire system. Therefore as a suggestion, Cloud Computing, Big Data, and the Internet of Things are the three main paradigms of industry 4.0 for improving and revolutionizing the healthcare system based on the information technology category, which managers can implement on the CSCM in the hospitals (Aceto et al., 2019; Lotfi et al., 2021a, 2021b, 2021c).

## Managerial insights and practical implications

It is necessary for managers not only to have goals but also to know how to use strategic and tactical decisions to achieve them. This study illustrates managerial insights for hospital managers. Since the importance of healthcare performance is increasing daily globally, managers need to know more about the crucial indicators to evaluate them as more valuable and accurate. Hence, the presented model provides some key performance indicators based on a healthcare circular supply chain. Managers, by understanding the relations among KPIs, can take better decisions. In other words, Managers can understand the importance of each indicator by using the results of this paper. Therefore, they can focus on the more valuable indicators. Cognitive mechanisms can lead to improving the system based on reducing risk management and providing corrective procedures, which is valuable for hospital managers.

There is no accepted agreement amongst scholars to consider healthcare KPIs influenced by different factors. This paper provided important KPIs based on the literature review and experts’ opinions. Hence, it has proposed a suitable framework to apply while evaluating the performance of CSCMs in the healthcare sector. In other words, the developed methodology for measuring HCSC management can be employed by hospital managers to evaluate and improve ICU efficiency. In addition, existing coordination and collaboration amongst supply chain members can be one of the appropriate strategies to adopt CSCM in hospitals (Bressanelli et al., 2019). Moreover, one of the important parts of implementing CSC practices is the ability to use the government’s capacity as the regulator (Govindan & Hasanagic, 2018).

## Conclusion and future recommendations

According to the importance of both circular supply chain and performance measurement recently, this study introduced an appropriate procedure for evaluating the healthcare circular supply chain, particularly in the ICU, which is one of the vital sectors of hospitals to survive patients. Although there are many types of research about performance measurement, evaluating the performance in the CSC has not been discovered. On the other hand, most of the papers illustrated KPIs without focusing on the relationships existing among them, which are useful to improve the internal healthcare supply chain. To fill the gap, this study provided a logical framework to introduce KPIs based on the previous studies and experts’ opinions and provided a diagram of their relationships which is used to rank the KPIs in the ICU based on CSC. After reviewing twenty-three papers twenty-nine indicators for evaluating the healthcare system were defined and based on the indicators’ frequency, those including more than 50% frequency were chosen. Thirteen indicators had illustrated by the literature review, then by using IFD methodology based on the experts’ opinion eight indicators were selected as KPIs in the circular supply chain of ICU. This study gathered data for FCM and Fuzzy DEMATEL. The CSC diagram based on FCM included twenty-two relationships among KPIs, and it was reduced to sixteen by incorporating Fuzzy DEMATEL. Consequently, inventory availability (C_1_), information availability (C_3_), and innovation and technology (C_6_) were more critical based on FCM-FDEMATEL results. Besides, the information technology group, which was defined in the discussion section, was the most influential category.

The presence of some limitations during the research is inevitable. For instance, data gathering based on online meetings with doctors and nurses, who were so busy during the pandemic, was not an effortless process. The implementation of the CSCM in some hospitals faces several barriers including a lack of collaboration and support between hospital managers. Despite the theoretical and practical contributions stated above, it is vital to acknowledge the limitations of our study that might offer opportunities for future research. First, our proposed framework based on Fuzzy MADM depends on the experts’ opinions. Consequently, it is recommended that experts’ opinions should be collected carefully or replaced with data-driven methods. Future research can employ statistical approaches or structural equation modelling (SEM) to complete or modify the list of KPIs in other fields, as well as to examine the possibility of generalizing the results. In addition, to resolve any doubts concerning human subjective judgments tools such as ANP or ISM, combining FCM with quantitative data/models, and grey system theory may be beneficial. In addition, diverse types of machine learning methods like a neural network, support vector machine, and linear regression can be applied to evaluate the weight of KPIs to improve performance. Furthermore, these methodologies can be integrated with grey-linear regression and nonlinear multivariable models, and so on to create robust prediction models. Additionally, the field of CSCM is developing rapidly. Therefore, it is necessary to update the literature review in a few years to keep up with the progress of the research field. Furthermore, researchers can focus more on the impact of some procedures to improve the healthcare system. For instance, the impact of industry 4.0 technologies such as machine learning, Industrial Internet of Things (IIoT) and artificial intelligence, etc., on the HCSC.
